# Drug-Related Deaths in China: An Analysis of a Spontaneous Reporting System

**DOI:** 10.3389/fphar.2022.771953

**Published:** 2022-02-25

**Authors:** Haona Li, Jianxiong Deng, Peiming Yu, Xuequn Ren

**Affiliations:** ^1^ Huaihe Hospital of Henan University, Kaifeng, China; ^2^ Adverse Drug Reaction Monitoring Center of Guangdong Province, Guangzhou, China; ^3^ School of Pharmacy, Henan University, Kaifeng, China

**Keywords:** adverse drug reactions, drug-related deaths, spontaneous reporting system, pharmacovigilance, pharmacoepidemiology

## Abstract

**Background:** Adverse drug reactions with an outcome of death represent the most serious consequences and are inherently important for pharmacovigilance. The nature and characteristics of drug-related deaths are to a large extent unknown in the Chinese population. This study aims to characterize drug-related deaths by analysis of individual case safety reports (ICSRs) with an outcome of death in China.

**Methods:** The characteristics of death ICSRs were analyzed by descriptive statistics of a large multi-provincial pharmacovigilance database in China.

**Results:** There were 1,731 ICSRs with an outcome of death, representing 0.95% of all serious cases and 0.05% of all reported ICSRs. Most death ICSRs (78.57%) were reported by medical institutions. Only 16.00% of death ICSRs were reported by manufacturers or distributors. The reporting rate of death ICSRs in the age group of 0–4 years was significantly higher than patients aged 5–64 years. Patients aged over 64 years had the highest reporting rate of death ICSRs. Male patients generally had a higher reporting rate of death ICSRs than female patients. However, the reporting rate of female patients exceeded that of male patients in the age group of 20–34 years. Among 3,861 drugs implicated, ceftriaxone sodium with 146 (3.78%) records of death ranked first. Dexamethasone with 131 (3.39%) records of death ranked second. Qingkailing, an injectable traditional Chinese medicine with 75 (1.94%) records of death, ranked the fifth most frequently implicated medicine.

**Conclusion:** Young children and elderly patients have a higher risk of drug-related deaths than patients aged 5–64 years. Female patients generally have a lower risk of drug-related deaths than male patients. However, female patients of reproductive age (aged 20–34 years) have a higher risk of drug-related deaths than male patients, hinting that physiological changes and drug uses for child bearing, giving birth, or birth control may significantly increase the risk of death for female patients aged 20–34 years. This paper suggests more research on the safe use of drugs for young children, elderly patients, and female patients of reproductive ages. Pharmacovigilance databases can be valuable resources for comprehensive understanding of drug-related problems.

## Introduction

Drugs are safe and effective therapies for numerous diseases and circumstances. The incidence of severe adverse effects is generally low in clinical use. However, once it happens, it may seriously threaten the health of patients and even lead to deaths (Moore et al., 2007; Lombardi et al., 2020). Adverse drug reactions (ADRs) have long been a major public health problem and a significant cause of morbidity and mortality worldwide (Lazarou et al., 1998; Pardo Cabello et al., 2016; Montané, et al., 2018; Pardo-Cabello et al., 2018; Patel and Patel, 2018; Montané and Castells, 2021). ADRs with an outcome of death represent the most serious consequences and are inherently important for pharmacovigilance (Marwitz et al., 2020). Although there have been studies assessing the incidence of drug-related deaths, information on the issue is rather limited, which left many questions unsolved (Montané et al., 2018).

Individual case safety reports (ICSRs) are an important information source for the study of ADRs (Klepper and Edwards, 2011; Streefland, 2018). Although spontaneous reporting data cannot give exact estimates of the magnitude of drug-related mortality, it can highlight large dimensions and some characteristics of the issue. There have been some studies on drug-related mortality by analysis of databases of spontaneous reporting in some countries (Wester et al., 2007; Leone et al., 2008; Marwitz et al., 2020). However, the nature and characteristics of drug-related deaths is to a large extent unknown in many developing countries including China.

Spontaneous reporting has the advantage of covering a large number of patients, a wide range of drugs, and being relatively cost-effective for the study of drug safety (Leone et al., 2008). With the development of a pharmacovigilance system in China, ICSRs with an outcome of death received each year have accumulated to a large volume and necessitate studies for better understanding of the sources and content. The objective of this study is to characterize ICSRs with an outcome of death by analysis of data sources of a large multi-provincial pharmacovigilance platform in China.

## Materials and Methods

### Data Source

The Chinese pharmacovigilance system is based on a network of 34 regional centers, including 23 provincial, 2 special administrative regional, 4 municipal, and 5 autonomous regional pharmacovigilance centers. Like many countries, ADR declaration is mandatory for hospitals, pharmaceutical manufacturers, and distributors but voluntary for patients in China. Adverse drug events (ADEs) reported spontaneously or derived from active pharmacovigilance projects or observational studies were evaluated by trained pharmacologists of regional pharmacovigilance centers and became complete ICSRs before entering pharmacovigilance databases. Data used in this study were retrieved from the Pan-pearl Delta ADR Monitoring Platform (Pan-pearl Platform), a multi-regional service of 12 member provincial pharmacovigilance centers in southern and central China to support postmarketing surveillance of medicinal products and promote cooperation between the regulatory authorities of 12 member provinces. Anonymized ICSRs from 12 member provincial pharmacovigilance centers were forwarded to the Pan-pearl Platform using a standardized format containing structured information. Each member provincial center was responsible for the management and quality control of the data.

Data stored in the platform consist of a modified national version of the World Health Organization Adverse Reaction Terminology (WHO-ART). This categorization is hierarchical and includes medical terms clustered into body system organ classes. Drugs are coded according to the WHO Collaborating Center for Drug Statistics Methodology International Anatomical Therapeutic Chemical (ATC) classification. The detailed description of the information resources of the Pan-pearl Platform is described elsewhere (Li et al., 2020). As of May 7, 2018, there were 3,429,002 ICSRs, including 182,417 serious ICSRs, available in the Pan-pearl Platform.

Concerning the data scale of the platform, there were 2,756,437 ICSRs in the Pan-pearl Platform from 2009 to 2017, accounting for 26.80% of all ICSRs reported to the spontaneous reporting system (SRS) of China during the same period (Li et al., 2018).

### Selection of Cases

There were 2,398 ICSRs with an outcome of death retrieved from the platform between January 1, 2002, and May 7, 2018, and 6,239 drug-ADE records obtained by splitting the 2,398 ICSRs off. The number of drug ADE records exceeded ICSRs because each ICSR may include more than one ADE and/or drug.

The causality between death and related drug(s) for each ICSR was assessed by the staff of a member provincial pharmacovigilance center based on the World Health Organization (WHO) standardized case–causality assessment criteria. ICSRs with causality assessments of unlikely, conditional/unclassified, or no documented causality assessments were excluded. There were 1,757 ICSRs with causality assessments of being certain (*N* = 48), probable (*N* = 376), or possible (*N* = 1,333).

**FIGURE 1:**
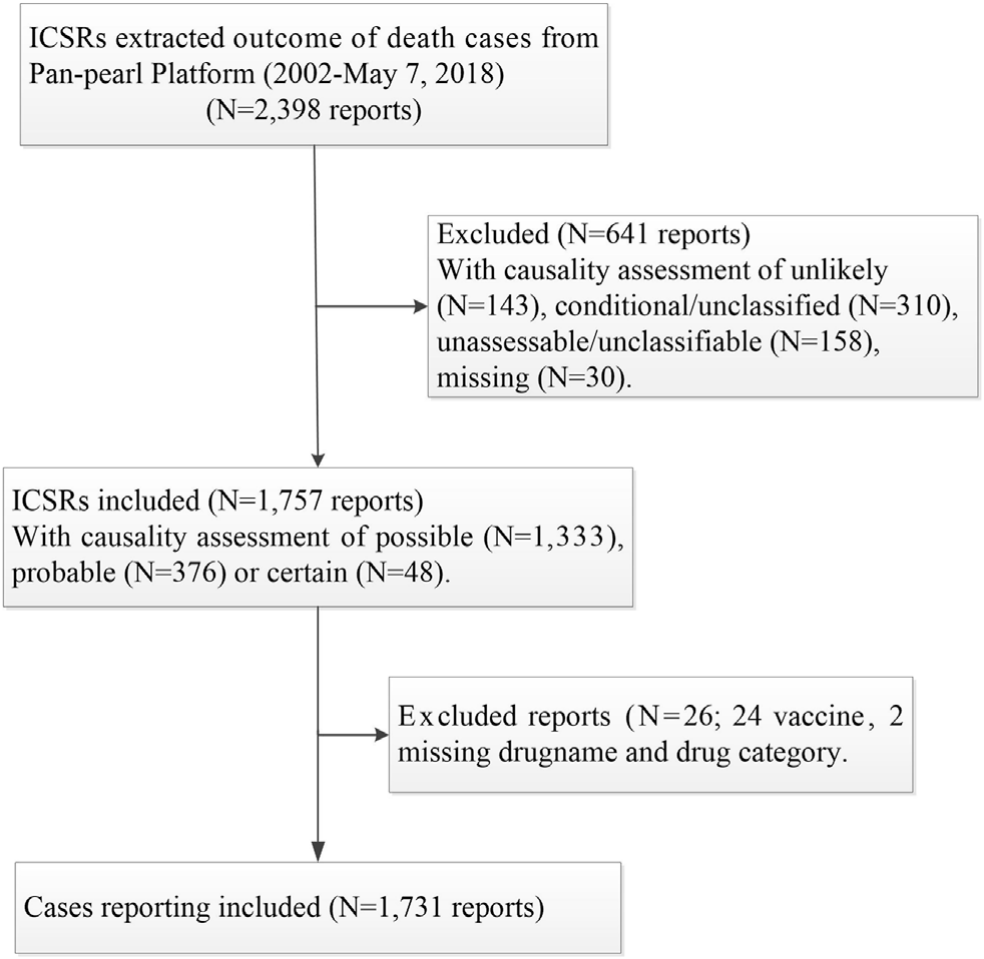
Flow chart of study design and case selection.

Two ICSRs with missed drug names and categories and 24 ICSRs related to vaccines were excluded. As most ICSRs of vaccines were generally handled by the National Disease Control and Prevention (NCDC), only a small proportion of ICSRs related to vaccines were reported to the Pan-pearl Platform. This study excluded ICSRs related to vaccines and left the issue to other studies designed for the safety of vaccines by putting the data from spontaneous reporting and the NCDC together. There were 1,731 ICSRs finally included for analysis. The case selection process and exclusion/inclusion criteria are summarized in Figure 1.

For the convenience of data processing and analysis, biologically inactive solutions used as solvents of other bioactive substances were excluded with the assumption that biologically inactive solutions (such as bi-distilled water or water solution containing glucose and/or sodium chloride) used as solvents of bioactive substances had made no contribution to the deaths of patients being treated. The number of ICSRs remained the same (1,731 ICSRs) after the exclusion of solvents.

### Data Analysis

Descriptive statistics were conducted to analyze the characteristics of death ICSRs, including annual numbers of ICSRs being forwarded to the platform and the proportion of ICSRs with an outcome of death in the platform, the geographical and time distribution, types of reporting sources, age and gender distribution, major ADEs related to death ICSRs and drugs or drug combinations implicated, dosage forms or routes of administration, frequently reported primary diseases, and so on. Due to the observational nature of the data and the expected existence of differences between groups, we did not perform statistical testing and only performed a comparative description.

## Results

### General Descriptions of Individual Case Safety Reports With an Outcome of Death

Among 3,429,002 ICSRs available in the Pan-pearl Platform between January 1, 2002, and May 7, 2018, there were 1,731 ICSRs with an outcome of death, representing 0.95% of all serious cases and 0.05% of all reported ICSRs. The annual number of death ICSRs exceeded 100 in 11 of the recorded 17 years from 2002 to 2018. Guangdong, Hunan, Yunnan, Guangxi, and Sichuan ranked top five provinces with highest numbers of death ICSRs among the 12 member provinces of the Pan-pearl region. The geographical and time distribution of death ICSRs in the platform is given in Supplementary Table S1.

**FIGURE 2:**
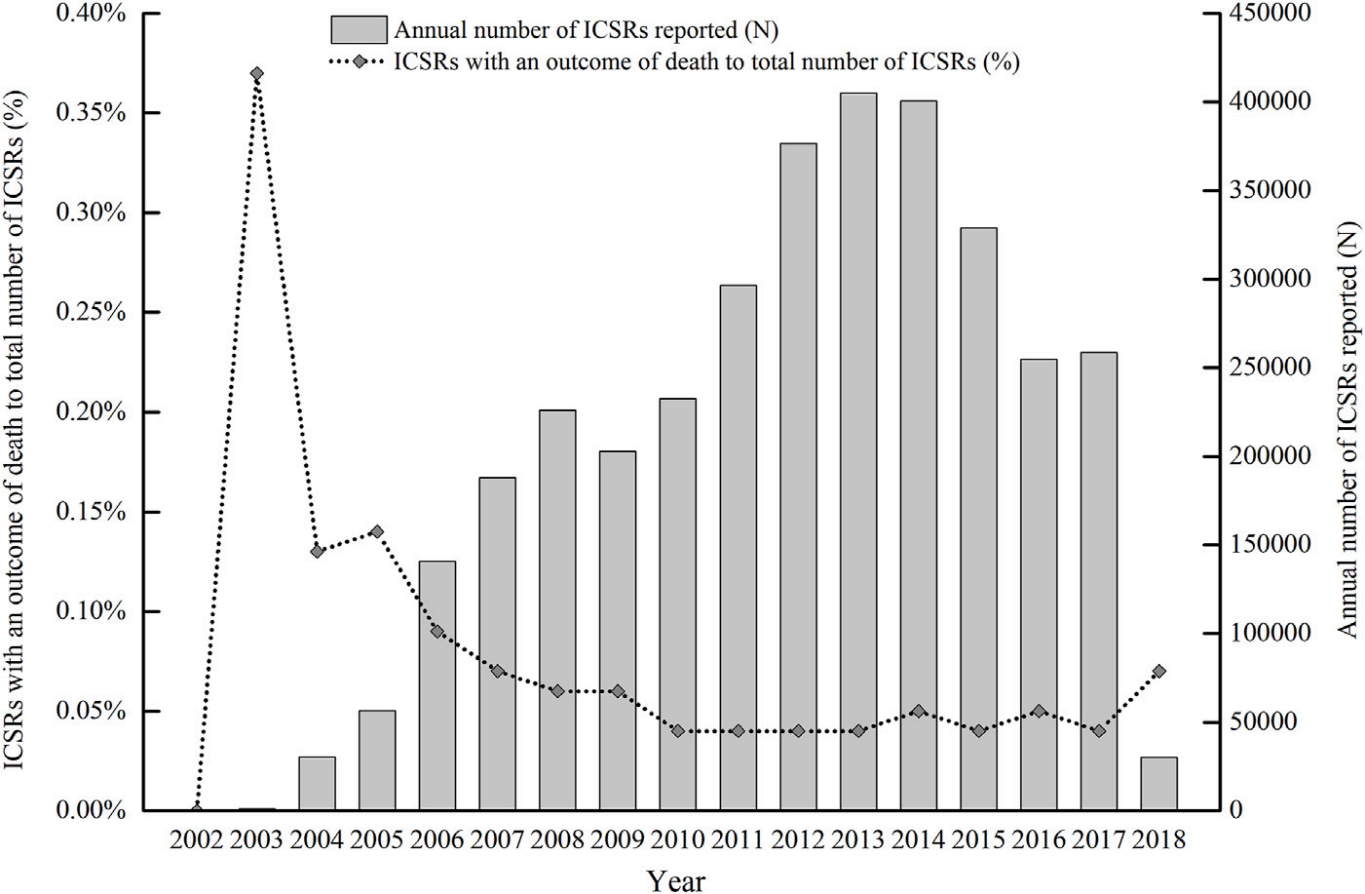
The annual numbers of ICSRs reported and the percentages of death ICSRs in Pan-pearl Platform.

The annual numbers of ICSRs being forwarded to the Pan-pearl Platform and the percentages of death ICSRs in the context of all ICSRs received annually are displayed in Figure 2.

With the development of the SRS in China, the annual numbers of ICSRs and ICSRs with an outcome of death were both on the rise and reached the peak in 2014. As a lot of provinces stopped uploading data to the platform, the annual numbers of ICSRs and ICSRs with an outcome of death started to decline after 2014. The platform officially ceased operation in 2019.

However, five provinces, including Fujian, Guangdong, Guanxi, Guizhou, and Hunan, continued to upload data throughout the whole period from 2002 to 2018. The total number of ICSRs reported of the five provinces was 2,029,916, accounting for 59.20% of the total number in the platform. The annual numbers of ICSRs reported and the percentages of death ICSRs in five provinces with all data available during the whole period are given in Supplementary Figure S1. The results are similar to the whole dataset of the platform.

Statistics on reporting sources of death ICSRs, including professions of the reporters and types of the reporting bodies, are given in Table 1. It can be seen that more than half of the death ICSRs were reported by physicians, followed by consumers (20.17%) and pharmacists (17.56%). Only 7.51% of the ICSRs were reported by nurses. As to the reporting bodies, medical institutions reported most death ICSRs (78.57%). Only 16.00% of death ICSRs were reported by manufacturers or distributors.

**TABLE 1:** Number of death ICSRs for different types of reporting sources.

Type of reporting sources	*N*	%
Type of reporters	—	—
Physicians	927	53.55
Consumers^a^	349	20.17
Pharmacists	304	17.56
Nurses	130	7.51
Missing	21	1.21
Type of reporting body	—	—
Medical institutions	1,360	78.57
Manufacturers	241	13.92
Consumers^b^	94	5.43
Distributors	36	2.08

a ICSRs, reported by consumers, including ICSRs, directly reported to pharmacovigilance centers by consumers or ICSRs, reported to drug manufacturers or distributors by consumers and transferred to pharmacovigilance centers.

b ICSRs, directly reported to pharmacovigilance centers by consumers.

### Age and Gender Distribution of Individual Case Safety Reports With an Outcome of Death

**FIGURE 3:**
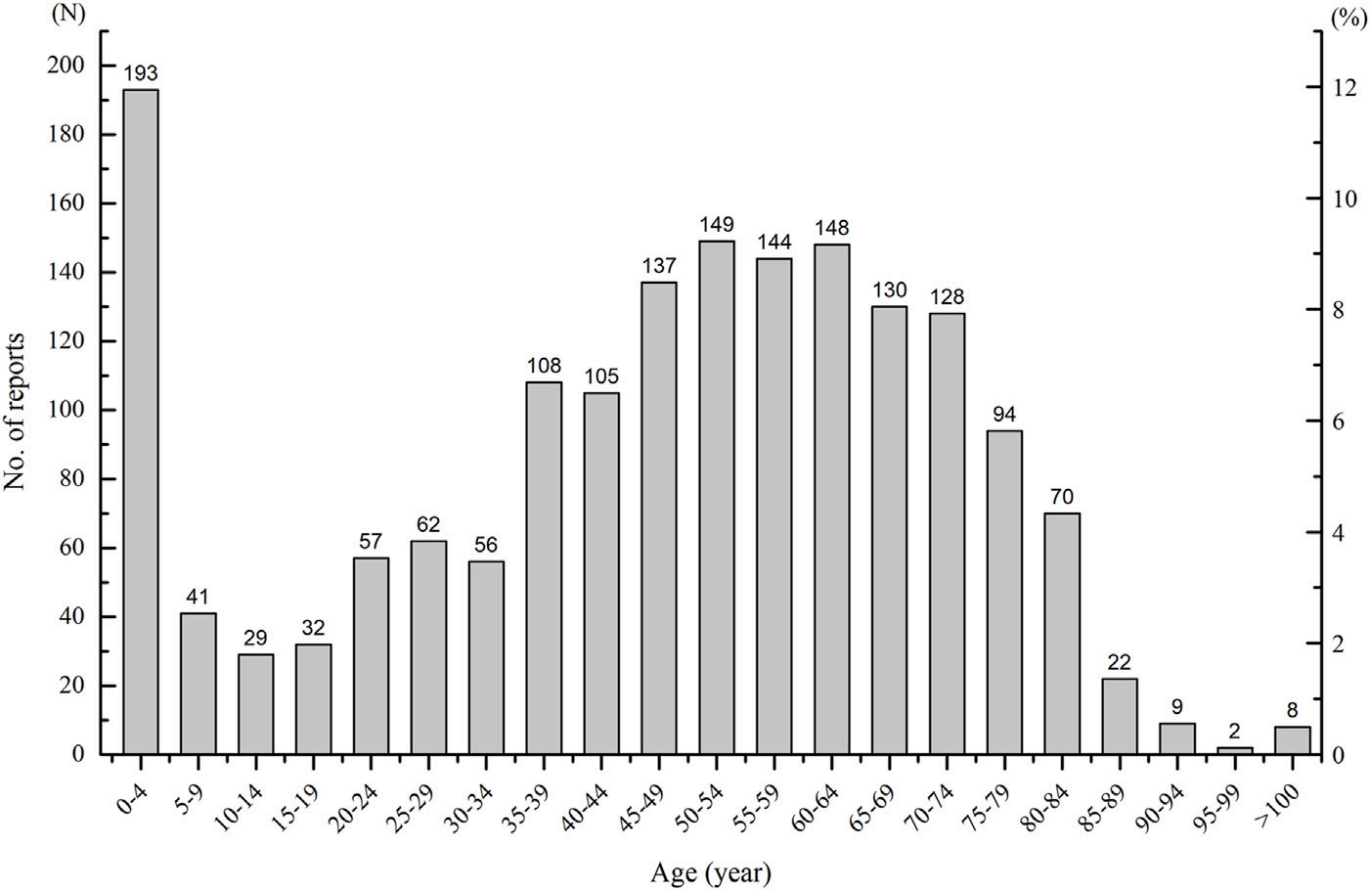
The numbers and percentages of reported death ICSRs for different age groups.

**FIGURE 4:**
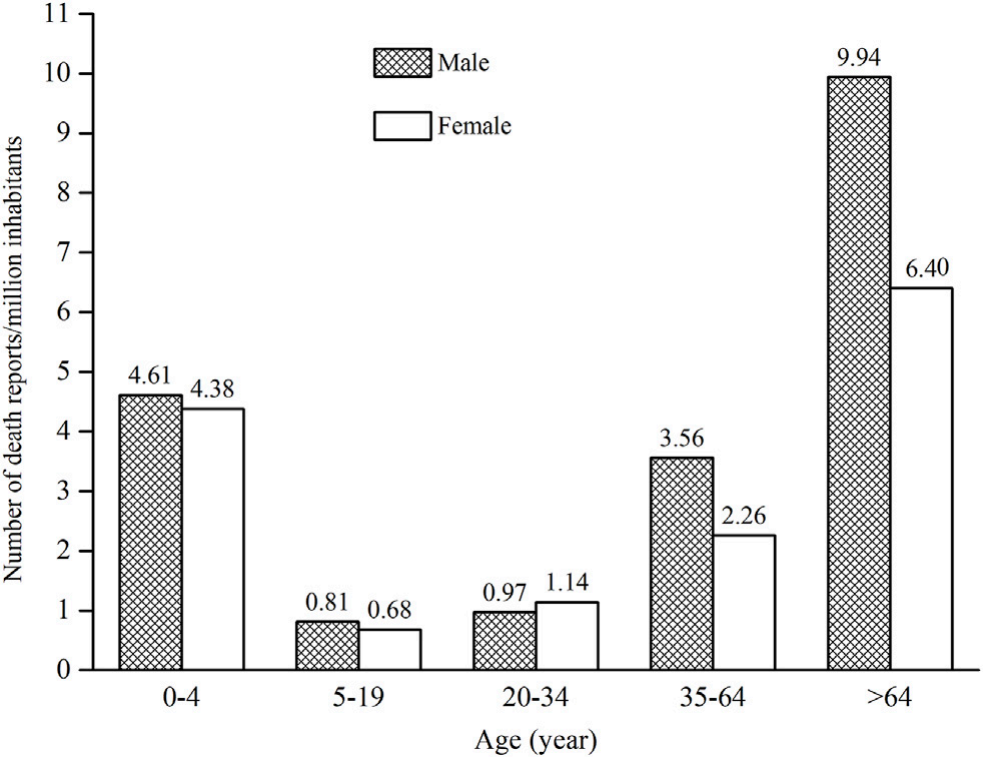
Age-gender distribution of death ICSRs by per million inhabitants.

**FIGURE 5:**
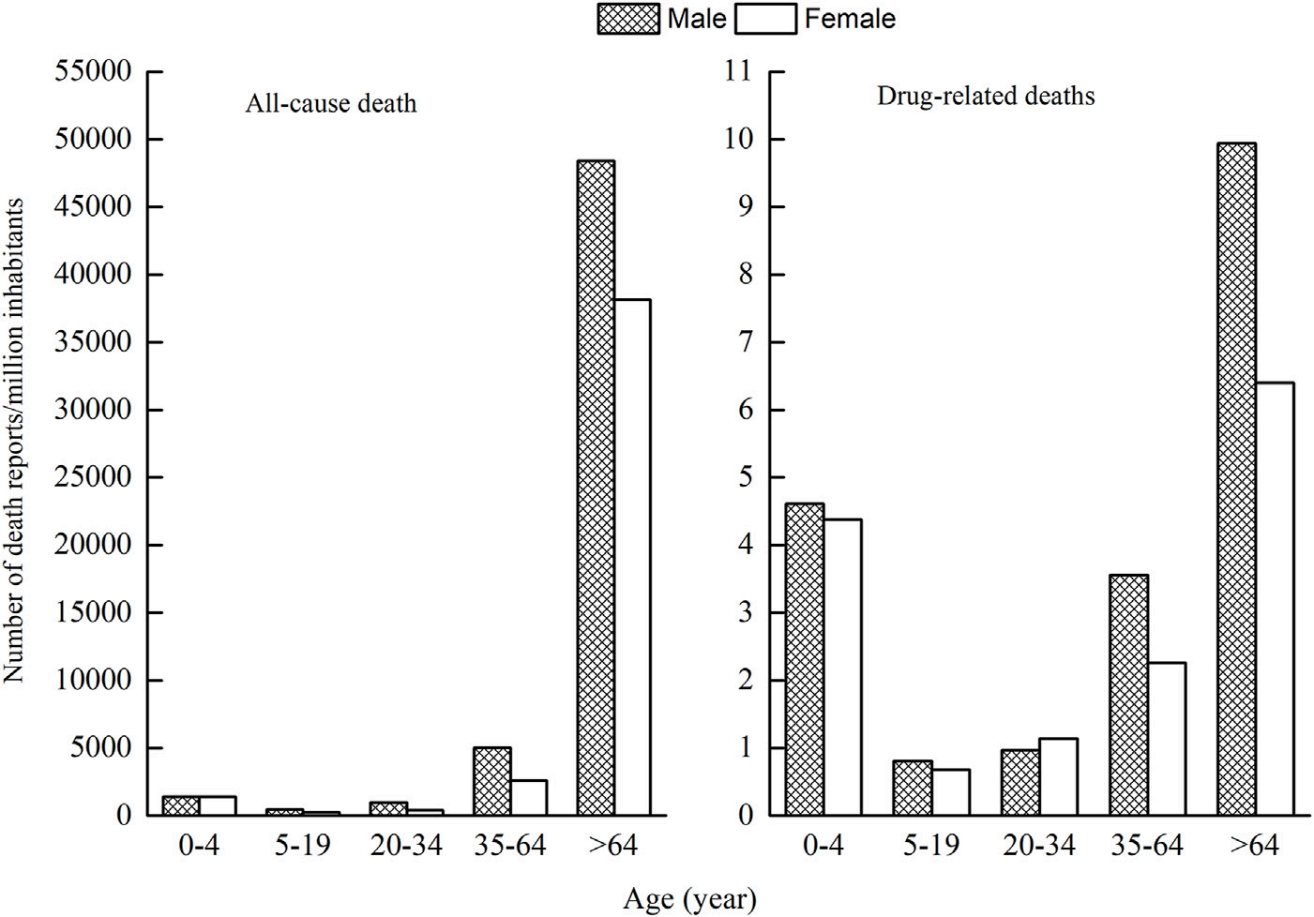
Age-gender distribution for all-cause deaths and drug-related deaths.

As ages were missing in seven ICSRs, the total number of ICSRs included in the analysis of age distribution is 1,724 (Figure 3). As can be seen in Figure 3, the age group of 0–4 years has the highest number of death ICSRs, accounting for 11.19% of all reported death ICSRs. The number of death ICSRs decreased with age and reached the lowest point at the age of 10–14 years. The numbers of death ICSRs increased with age for patients aged over 14 years and began to decline in patients aged over 65 years. Since there were significant differences in the sizes of the population between different age groups and the number of death ICSRs may be influenced accordingly, this study standardized the age–gender distribution of death ICSRs by per million inhabitants to strip out the impact of population sizes between different age groups. As there were 12 ICSRs with missing age or gender, the total number of ICSRs included in the analysis of age–gender distribution is 1,719 (Figure 4 and Figure 5).

Since all-cause mortality may influence the age–gender distribution of death ICSRs, we calculated all-cause deaths and drug-related deaths of both sexes for different age groups (Figure 5). Data of all-cause mortality come from annual census figures of the National Bureau of Statistics of China (NBS) (National Bureau of Statistics of China, 2010).

Comparing the age–gender distribution of all-cause deaths and drug-related deaths, there were 4.50 drug-related deaths per million inhabitants for the age group 0–4 years, accounting for 11.23% of the total deaths of all age groups. The number of all-cause deaths per million inhabitants in the age group 0–4 years was 1,418.80, accounting for 1.63% of the total deaths of all age groups.

From Figures 4 and 5, it can be seen that female patients generally have a lower number of death ICSRs than male patients for almost all age groups except for the age group of 20–34 years. For better understanding the reason why the number of death ICSRs of female patients exceeded male patients at the age group of 20–34 years, this study extracted detailed information on death ICSRs related to drug use of female reproductive disorders from the dataset and presented these in Supplementary Table S2.

### ADEs Frequently Related to Death Individual Case Safety Reports and Drugs Implicated

This study explored ADEs frequently implicated in death ICSRs and suspected drugs related to the specific ADEs (Supplementary Table S3). Anaphylactic shock is the most common ADE implicated in death ICSRs.

**FIGURE 6:**
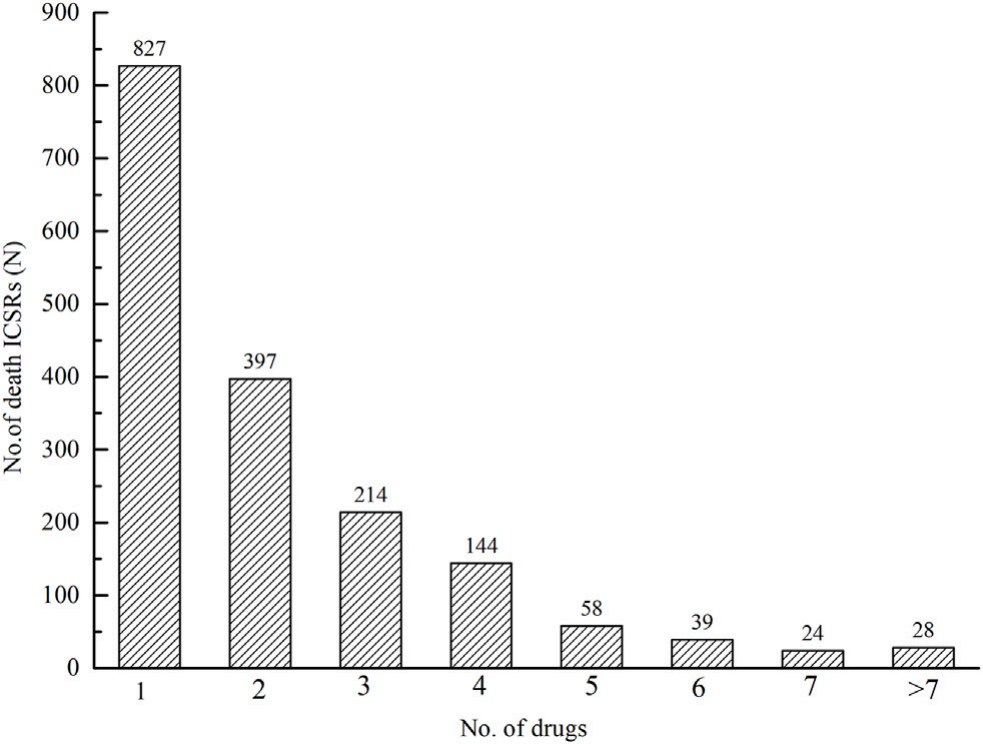
The number of suspected drugs implicated and the corresponding number of death ICSRs.

There were 3,861 drugs implicated in 1,731 ICSRs with an outcome of death. Among 1,731 ICSRs, there were 827 (47.78%) ICSRs with one drug suspected, 397 (22.93%) ICSRs with two drugs suspected, 214 (12.36%) with three, and 144 (8.32%) with four (Figure 6).

This study also analyzed drugs frequently implicated in death ICSRs and the corresponding high-frequency ADEs related to the specific drug (Table 2). Among 3,861 drugs implicated, anti-infectious agents were drugs most frequently implicated, including 146 (3.78%) cases of ceftriaxone sodium, 100 (2.59%) cases of ribavirin, 99 (2.56%) cases of cefoperazone sodium and sulbactam sodium, 58 (1.50%) cases of cefotaxime sodium, 56 (1.45%) cases of clindamycin, 53 (1.37%) cases of benzylpenicillin, and 52 (1.35%) cases of potassium sodium dehydroandrograpolide succinate. Dexamethasone with 131 (3.39%) cases of death ICSRs ranked second. Qingkailing, an injectable traditional Chinese medicine (TCM), with 75 (1.94%) cases of death ICSRs ranked the fifth most frequently implicated medicine.

**TABLE 2:** Drugs frequently implicated and the corresponding high-frequency ADEs.

Rank	Drugs suspected	N (%) of records (*n* = 3,861)^a^	High-frequency ADEs (IT)^b^ (frequencies of records)^c^
1	Ceftriaxone sodium	146 (3.78)	Anaphylactic shock (77), death (24), anaphylactoid reaction (9), dyspnea (8), sudden death (5), tics (4), hyperpyrexia (3), anaphylactic reaction (3), asystolia (3)
2	Dexamethasone	131 (3.39)	Anaphylactic shock (62), dyspnea (19), death (16), anaphylactoid reaction (9), cyanosis (6), vomiting (4), shock (4), anaphylactic reaction (3), respiratory failure (3)
3	Ribavirin	100 (2.59)	Anaphylactic shock (40), death (12), dyspnea (11), tics (5), anaphylactoid reaction (5), respiratory failure (5), sudden death (5), hyperpyrexia (5), rigors (4)
4	Cefoperazone sodium and sulbactam sodium	99 (2.56)	Anaphylactic shock (62), dyspnea (11), death (6), cyanosis (6), sudden death (5), tics (4), anaphylactoid reaction (4), pruritus (4), chest distress (3)
5	Qingkailing^d^	75 (1.94)	Anaphylactic shock (40), death (13), tics (5), anaphylactoid reaction (5), dyspnea (5), hyperpyrexia (2), anaphylactic reaction (2), rigors (2), laryngeal edema (2)
6	Vitamin C	71 (1.84)	Anaphylactic shock (34), death (10), dyspnea (6), rigors (5), tics (3), fever (3), respiratory failure (3), erythema multiforme (2)
7	Cefotaxime sodium	58 (1.50)	Anaphylactic shock (38), death (4), anaphylactoid reaction (3), tics (2), anaphylactic reaction (2), dyspnea (2), vomiting (2)
8	Clindamycin	56 (1.45)	anaphylactic shock (23), death (10), dyspnea (5), anaphylactoid reaction (5), sudden death (3), laryngeal edema (2), respiratory failure (2)
9	Benzylpenicillin	53 (1.37)	Anaphylactic shock (32), death (6), anaphylactoid reaction (5), dyspnea (4), respiratory failure (3), chest distress (3)
10	Potassium sodium dehydroandrograpolide succinate	52 (1.35)	Anaphylactic shock (22), dyspnea (8), death (6), tics (3), anaphylactoid reaction (3), anaphylactic reaction (2), vomiting (2), respiratory failure (2), asystolia (2)
10	Vitamin B_6_	52 (1.35)	Anaphylactic shock (24), death (8), dyspnea (5), tics (3), rigors (3), erythema multiforme (2), fever (2), asystolia (2), chest distress (2)

Note: As more than one drug and/or ADE may be implicated in one ICSR, the total frequencies exceed the total number of ICSRs.

a There were 3,861 drug death records derived from 1,731 ICSRs.

b IT, WHO-ART, included terms

c Adverse events with at least two records

d A widely used traditional Chinese medicine injection; others are conventional medicines.

Suspected drug combinations frequently implicated in death ICSRs and corresponding high-frequency ADEs related to the specific drug combination are given in Table 3.

**TABLE 3:** Suspected drug combinations frequently implicated and corresponding high-frequency ADEs.

Drug combinations suspected	N (%) of ICSRs (*n* = 904)^a^	Corresponding ADEs (IT) (no. of ICSRs)
Dexamethasone sodium phosphate injection + cefoperazone sodium and sulbactam sodium injection	7 (0.77)	Anaphylactic shock (6), dyspnea (1), cyanosis (1)
Dexamethasone sodium phosphate injection + ceftriaxone sodium injection	6 (0.66)	Anaphylactic shock (3), anaphylactoid reaction (2), death (1)
Dexamethasone sodium phosphate injection + lincomycin hydrochloride injection	4 (0.44)	Anaphylactic shock (2), death (2), asystolia (1), shock (1)
Dexamethasone sodium phosphate injection + ceftriaxone sodium injection + qingkailing injection^b^	4 (0.44)	Anaphylactic shock (2), tics (1), hyperpyrexia (1), rigors (1), death (1)

IT, WHO-ART, included terms.

a There were 904 ICSRs, with concomitant use of two or more drugs

b A widely used traditional Chinese herbal injection; others are conventional medicines.

This study explored the number of drugs suspected and the corresponding number of drug death records for conventional medicines and TCMs (Table 4). Among the 3,861 drugs implicated in death ICSRs, there were 3,357 conventional medicines and 504 TCMs. TCMs accounted for 13.05% of the total number of drugs implicated.

**TABLE 4:** Drug death records for conventional medicines and TCMs.

No. of drugs suspected	Conventional medicine, no. (%) of records	TCMs, no. (%) of records	Total, no. (%) of records
1	739 (89.36)	88 (10.64)	827 (100)
2	694 (87.41)	100 (12.59)	794 (100)
3	562 (87.54)	80 (12.46)	642 (100)
4	495 (85.94)	81 (14.06)	576 (100)
5	250 (86.21)	40 (13.79)	290 (100)
6	209 (89.32)	25 (10.68)	234 (100)
7	143 (85.12)	25 (14.88)	168 (100)
≥8	265 (80.30)	65 (19.70)	330 (100)
Total	3,357 (86.95)	504 (13.05)	3,861 (100)

Note: Number refers to drug death records and exceeds the number of death ICSRs, as two or more suspected drugs may be implicated in each ICSR.

### Dosage Forms, Routes of Administration, and Primary Diseases Reported

Major dosage forms implicated in death ICSRs for conventional medicine and TCM are given in Table 5. For conventional medicines, there were 2,877 (85.70%) drugs in the dosage form of injections, while the number (percentage) of injections for TCMs is 388 (76.98%). Injections were the dosage form ranked first in death ICSRs for both conventional medicines and TCMs. Traditional Chinese herbal decoction pieces, as the main forms of TCM products, ranked second followed by injections.

**TABLE 5:** Dosage forms implicated in death ICSRs.

Dosage forms	Conventional medicine, *N* (%)	TCM, *N* (%)
Injection	2,877 (85.70)	388 (76.98)
TCM decoction pieces	**—**	36 (7.14)
Tablet	329 (9.80)	24 (4.76)
Capsule	78 (2.32)	19 (3.77)
Granule	13 (0.39)	17 (3.37)
Others^a^	60 (1.79)	20 (3.97)
Total, *n* (%)	3,357 (100.00)	504 (100.00)

Note: Number refers to drug death records.

a Others include inhalants, oral liquids, dropping pills, pills, ointments, suppository, eye drops, gels, etc.

In order to explore the impact of routes of administration to the outcome of death, we calculated the number of cases of different routes of administration in death ICSRs (Table 6). As can be seen from Table 6 there are some similarities between conventional medicines and TCMs. Among all routes of administration, intravenous injections ranked first, followed by oral dosage forms and intramuscular injections. As there were injections other than intravenous injections, such as intramuscular injection, peritoneal injection, subcutaneous injection, intradermal injection, and so on, the number of death ICSRs for all injections accounted for more than 80% of ICSRs of all dosage forms, reflecting that physicians in China are prone to prescribe drugs parenterally.

**TABLE 6:** Routes of administration implicated in death ICSRs.

	Intravenous injection, *n* (%)	Oral, *n* (%)	Intramuscular injection, *n* (%)	Intraperitoneal injection, *n* (%)	Hypodermic injection, *n* (%)	Inhalation, *n* (%)	Other, *n* (%)	Total row, *n* (%)
Conventional medicine	2,505 (74.62)	417 (12.42)	199 (5.93)	53 (1.58)	47 (1.40)	30 (0.89)	106 (3.16)^a^	3,357 (86.95)
TCM	364 (72.22)	109 (21.63)	26 (5.16)	0 (0.00)	0 (0.00)	0 (0.00)	5 (0.99)^b^	504 (13.05)
Total column, *n* (%)	2,869 (74.31)	526 (13.62)	225 (5.83)	53 (1.37)	47 (1.22)	30 (0.78)	111 (2.87)	3,861 (100.00)

Note: Number refers to drug death records.

a Intracutaneous injection, pump injection, artery injection, intrathecal injection and so on.

b Sublingual administration, external use and local drug delivery.

This study analyzed frequently reported primary diseases implicated in death ICSRs (Table 7). In the dataset, primary diseases refer to illnesses that need to be diagnosed or treated. They are usually equivalent to therapeutic indications (reasons for use) of the suspected drugs but not always the case. For example, iohexol may be used for radiological examination (reason for use) for a patient with pulmonary malignancy as primary disease. Acute upper respiratory tract infections ranked the first primary disease, followed by chronic renal failure (N18), influenza, virus not identified (J11), acute bronchitis (J20), and so on. Eight of the top 10 primary diseases in death ICSRs can be classified as infectious diseases, reflecting the high incidence of infectious diseases in the Chinese population and the high frequency of drug use and deaths.

**TABLE 7:** Most frequently reported primary diseases (top 10).

Rank	Disease code (ICD-10)^a^	System code (ICD-10)	N	%
1	Acute upper respiratory infections of multiple and unspecified sites (J06)	Diseases of the respiratory system	118	6.82
2	Chronic renal failure (N18)	Diseases of the genitourinary system	38	2.20
3	Influenza, virus not identified (J11)	Diseases of the respiratory system	32	1.85
4	Acute bronchitis (J20)	Diseases of the respiratory system	31	1.79
5	Pneumonia, organism unspecified (J18)	Diseases of the respiratory system	28	1.62
6	Other forms of acute ischemic heart disease (I24.8)	Diseases of the circulatory system	19	1.10
7	Bronchopneumonia, unspecified (J18.0)	Diseases of the respiratory system	18	1.04
8	Other gastroenteritis and colitis of infectious and unspecified origin (A09)	Certain infectious and parasitic diseases	16	0.92
9	Unspecified chronic bronchitis (J42)	Diseases of the respiratory system	15	0.87
10	Asthma, unspecified (J45.9)	Diseases of the respiratory system	13	0.75

a ICD-10, International Statistical Classification of Diseases and Related Health Problems 10th Revision, 2016, while disease statistics excluded co-morbid conditions.

## Discussion

### Reporting Sources of Death Individual Case Safety Reports

This study provided information on the characteristics of drug-related deaths in China between 2002 and 2018. Statistics showed that physicians ranked first in the reporting of death ICSRs (53.55%), and pharmacists ranked second (17.56%). The role and knowledge structure of pharmacists are advantageous for reporting ADRs (Barnes and Butler, 2020). As to the reporting bodies, 78.57% of death ICSRs were reported by medical institutions. Only 16.0% of death ICSRs were reported by manufacturers or distributors. This is very different from similar studies in the US demonstrating that most ICSRs (96.20%) came from manufacturers (Marwitz et al., 2020). China's SRS system lacks active participation of drug manufacturers and distributors. The awareness and reporting capacity of manufacturers and distributors need to be improved.

### Percentage of Death Individual Case Safety Reports in the Context of Individual Case Safety Reports Reported in the SRS System

With the development of the SRS in China, the annual numbers of ICSRs and ICSRs with an outcome of death were both on the rise. It is noteworthy that there was an exceptionally high proportion of death ICSRs between 2003 and 2006, and the percentages of annual death ICSRs showed a downward trend during the early years of China's SRS system from 2002 to 2010. This can be explained by the characteristics of the initial stage of the SRS of China. More attention was paid to reports of serious cases and deaths at the initial stage, and the total number of annual reports was small. After 2010 the percentages of death ICSRs went up and down in a narrow range, reflecting that the number of death ICSRs increased proportionally with the total number of ICSRs reported except in the year 2018 in which only a small number of total ICSRs and death ICSRs were uploaded to the platform.

There is also another influencing factor added to this trend. The supervisory authorities have been pushing hard on medical institutions, pharmaceutical manufacturers, and distributors to report more ICSRs, resulting in more reports of minor ADEs or external symptoms of related physical conditions reported and extra decrease in the proportion of serious or death ICSRs after 2010 (Li et al., 2019).

Death ICSRs in the present study represented 0.05% of all reported ICSRs, much less than previous similar studies in other countries, such as approximately 1.00% in Canada (Gowdey and Brennan, 1985), 2.40% and 3.10% in Sweden (Böttiger et al., 1979; Wester et al., 2007), 1.66% in Italy (Leone et al., 2008), and 6.30% and 9.60% in the US (Chyka, 2000; Marwitz et al., 2020). The gap in the proportion of death ICSRs between China and other countries provides a new clue for reconsidering the policies governing ADE reporting.

### Characteristics of Age and Gender Distribution of Death Individual Case Safety Reports

The nonstandardized age and gender distribution of ICSRs with an outcome of death may not accurately reflect the true nature of death ICSRs because of the significant differences in the sizes of population between different age groups. This study standardized the age–gender distribution of death ICSRs by calculations of the number of death ICSRs per million inhabitants to strip out the impact of population sizes. The number of death ICSRs per million inhabitants in the age group of 0–4 years was significantly higher than patients aged 5–64 years. The lowest point was at the age group of 5–20 years, and then the number increased continuously with age. Patients aged over 64 years have the highest number of death ICSRs per million inhabitants.

A number of reasons may contribute to the exceptionally high reporting number of death ICSRs per million children aged 0–4 years. For very young children, the variable weight and body surface area, and immaturity of the organ system, may affect their ability to metabolize and excrete medications (Venkatesh, 2010). Research has established marked differences between children and adults in drug pharmacokinetics and pharmacodynamics (Zimmerman et al., 2019). Many medicines prescribed to children have not been studied in this patient population or formally approved for pediatric use (Roberts et al., 2003; Hwang et al., 2019; Zimmerman et al., 2019). Besides, a lot of medications lack standardized dosing regimens for young children (Rauch et al., 2018; Powell et al., 2021) and make the appropriate use of drugs for very young children difficult and cause more adverse events to occur. The immaturity of the organ system of very young children may cause more serious events or deaths.

Comparing the age–gender distribution between drug-related deaths and all-cause deaths, the percentage of drug-related deaths for the age group of 0–4 years relative to all age groups is significantly higher than that of all-cause deaths, hinting that children aged 0–4 years are more vulnerable to drug-related deaths. The safety use of medicine in young children calls for more attention and scientific research.

The high rate of drug-related deaths for patients aged over 64 years is expected and in agreement with other studies on drug-related mortality (Chyka, 2000; Wester et al., 2007; Hartholt et al., 2010; Ruiter et al., 2012). It has been reported that the percentage of hospitalizations attributed to drug therapy is highest for elderly people (Beijer and de Blaey, 2002; Pirmohamed et al., 2004; van der Hooft et al., 2006; Salvi et al., 2012; Linkens et al., 2020). The reason can be explained by a series of age-related changes in pharmacokinetics and pharmacodynamics. The body function deteriorates with age for elderly people; the frequency and severity of underlying diseases and frequency of drug uses or combined drug uses (polypharmacy and risk of drug-drug interactions) are likely to increase with age (Letinier et al., 2019; Kardas et al., 2021). All these may contribute to the high reporting frequencies of total ICSRs and death ICSRs in elderly people.

In terms of gender, the number of death ICSRs for male patients was generally higher than for female patients (58.52 vs. 41.31%), hinting that male patients generally have a higher risk of death ICSRs than female patients. The results are in agreement with the fact that men have higher all-cause mortality than women in general and in every age group.

Female patients usually take more drugs (Ferrajolo et al., 2019) and tend to experience more ADEs than male patients do (Wester et al., 2007; de Vries et al., 2019). However, the number of death ICSRs of female patients was generally lower than male patients. The contradiction has been studied, and the results are generally in agreement with similar studies (Chyka, 2000; Zoppi et al., 2000; Buajordet et al., 2001; Juntti-Patinen and Neuvonen, 2002).

Opposite to the general rule, the age group of 20–34 years is the only age group in which the number of death ICSRs of female patients outnumbered male patients. This may be explained by the fact that female patients aged 20–34 years may experience physiological changes and drug uses for child bearing, giving birth, or birth control and also possibly higher susceptibility to drug-related injuries than male patients at this age. More studies are needed for a better understanding of the safety of medicinal products for female patients aged 20–34 years.

### Drugs, Dosage Forms and ADEs Implicated in Death Individual Case Safety Reports

This study demonstrated that there were a variety of drugs associated with death ICSRs, indicating that drugs of various pharmacological groups may cause fatalities. Anti-infectious agents, including ceftriaxone sodium, ribavirin, cefoperazone sodium and sulbactam sodium, cefotaxime sodium, clindamycin, benzylpenicillin, and potassium sodium dehydroandrograpolide succinate, ranked top 10. Injections were the most frequent dosage form, and anaphylactic shock was the most frequently reported ADE. The results were similar to an Italian study (Leone et al., 2008). Anaphylactic shock is a known serious ADE to a series of anti-infectious agents, and the frequency depends on both the population exposed and the dosage forms or routes of administration (Baldo et al., 2001). Like what happened in Italy (Vaccheri et al., 2000; Leone et al., 2008) widespread administering of anti-infectious agents parenterally is also a serious problem in China (Long and Li, 2010; Zhang, 2010). Dexamethasone with 131 (3.39%) cases of death ICSRs ranked second.

There are some differences between our study and some other similar studies. There were studies reporting that blood and bone marrow dysfunction and hemorrhages were the most frequently reported ADEs (Wester et al., 2007; Leone et al., 2008). Anticoagulants were most frequently involved in spontaneous fatal reports in a German study (Tiaden et al., 2005) and a Swedish study (Wester et al., 2007). Anticoagulants were among the most frequently implicated drug classes in some Nordic studies investigating fatal ADRs in hospital settings (Zoppi et al., 2000; Ebbesen et al., 2001). Antineoplastic and immunosuppressive agents were the drugs most frequently suspected in spontaneously reported deaths in a US study (Chyka, 2000). Nervous system agents were the most frequently reported medications associated with death ICSRs, followed by anti-infective agents for systemic use, musculoskeletal and cardiovascular system agents, and musculoskeletal agents in two Canadian studies (Mittmann et al., 1997; Liu et al., 2001). It is difficult to explain these differences, but they may be due to differences in drug utilization patterns or different attitudes of the patients and health professionals towards ADR reporting. It is necessary to further explore the correlation between drug utilization patterns and health results.

Concerning drug combinations suspected, dexamethasones combined with various anti-infectious agents, including cefoperazone sodium and sulbactam sodium injection, ceftriaxone sodium injection, lincomycin hydrochloride injection, ceftriaxone sodium injection, and Qingkailing injection, were the most frequently implicated drug combinations in death ICSRs. The results indicate the possibility of misuse or overuse of the combination of glucocorticoids and anti-infectious agents in China which require for more attention and research.

It is noteworthy that Qingkailing, an injectable TCM which has been reported in widespread use in China (Li et al., 2018), ranked the fifth most frequently implicated medicine in death ICSRs. Qingkailing combined with dexamethasone sodium phosphate injection and ceftriaxone sodium injection ranked the fourth most frequently implicated drug combination. In China's SRS system, Qingkailing is among the most frequently reported TCM products, and a warning of severe adverse effects has been issued by the Food and Drug Administration of China (CFDA) (Li et al., 2015; Li et al., 2018), the predecessor of the National Medical Products Administration (NMPA).

TCMs accounted for 13.05% of the total number of drugs implicated in death ICSRs, reflecting the widespread use and prevalence of serious adverse effects of TCM products.

It is generally believed by many people in China that TCMs, being natural or derived from natural products, are safe remedies with no harmful effects. Statistics in this study showed that the risk of adverse effects of TCM products cannot be ignored. The risk of adverse effects of TCM injections, like conventional injections, is much higher than any other dosage forms (Li et al., 2019). The safety surveillance of TCM injections calls for more attention and research (Li et al., 2019).

Infectious diseases were the most frequently reported primary diseases implicated in death ICSRs, reflecting the high incidence of the diseases and drug use for anti-infectious purpose and the large scale of mortality related to anti-infectious medicines. In addition to infectious diseases, chronic renal failure and other forms of acute ischemic heart disease were also frequently related to death ICSRs.

### Limitation

We should bear in mind several important limitations. Firstly, this study is based on spontaneously reported ICSRs, which mainly reflect the concerns of healthcare professionals rather than the actual number of deaths. Other shortcomings related to spontaneous reporting may also affect the reliability of the study, such as the quality of the information, selective reporting, and under-reporting (Guo et al., 2021).

Secondly, the correlation between death and drug use was based on the judgments of the reporters and specialists of provincial pharmacovigilance centers of the Pan-pearl region. As a significant proportion of patients whose deaths were attributed to a drug were terminally ill, it would be a complicated task to identify the drugs as the cause of death, especially in patients with polypharmacy and comorbidity. There were always plenty of other possible explanations which cannot be ruled out. Moreover, in fatal cases with a long period of disease, it is more likely that clinicians attribute deaths to underlying diseases than to the therapies provided.

Thirdly, the limitation on the scope of the data source cannot be ignored. The dataset being used in the study were retrieved from a pharmacovigilance platform with 12 member provincial centers in southern or central China, accounting for 26.80% of all ICSRs reported to the SRS of China. This study mainly reflects the scales and features of drug-related deaths in southern and central China. Generalization of the results to the whole Chinese population should be done with caution.

Last but not the least, this study ignored the effect of inactive solutions used as solvents of bioactive substances that might affect the validity of the results to some degree as biologically inactive solutions might have some effect themselves or influence the effects of the drugs being solved and therefore influence the outcome of the patient.

## Conclusion

Children aged 0–4 years and patients aged over 64 years were at higher risk of drug-related deaths than patients aged 5–64 years. The reporting rates of drug-related deaths of female patients were generally lower than male patients except for the age group of 20–34 years, hinting that the physiological changes and drug uses for child bearing, giving birth, or birth control may significantly increase the risk of death for female patients aged 20–34 years. Anti-infectious agents were the drugs most frequently implicated in drug-related deaths. Dexamethasone ranked second. The dosage form of injections, primary diseases of infectious diseases, and ADE of anaphylactic shock were most frequently implicated in drug-related deaths. TCMs accounted for 13.05% of the total number of drugs implicated. The safety use of drugs for very young children, elderly patients, and female patients of reproductive ages calls for more attention and research. Pharmacovigilance databases can be valuable resources for comprehensive understanding of drug-related problems.

## Data Availability

The original contributions presented in the study are included in the article/Supplementary Material; further inquiries can be directed to the corresponding author.
