# Supplementary material for: Drug-Related Deaths in China: An Analysis of a Spontaneous Reporting System
Source: Front Pharmacol. 2022 Feb 25;13:771953. doi: 10.3389/fphar.2022.771953 (PMC8914085; doi:10.3389/fphar.2022.771953)
Supplement: Supplementary file 1 [file DataSheet1.pdf]

Supplementary Material

Drug-Related Deaths in China: an Analysis of a Spontaneous Reporting System

Haona Li PhD[1]*, Jianxiong Deng PhD[2], Peiming Yu PhD[3], Xuequn Ren PhD[1]

[1]Huaihe Hospital of Henan University, Kaifeng, Henan, China

[2]Adverse Drug Reaction Monitoring Center of Guangdong Province, Guangzhou, Guangdong, China

[3]School of Pharmacy, Henan University, Kaifeng, Henan, China

***Corresponding author:**

Haona Li

haonali@vip.henu.edu.cn

Key words: adverse drug reactions, drug-related deaths, spontaneous reporting system, pharmacovigilance, pharmacoepidemiology.

Supplementary Table 1. Geographical and time distribution of ICSRs with an outcome of death in the Pan-pearl Platform

Province	2002	2003	2004	2005	2006	2007	2008	2009	2010	2011	2012	2013	2014	2015	2016	2017	2018	Total (%, n=1731)
Fujian	0	0	1	9	4	18	7	7	2	2	2	7	2	6	3	5	0	75 (4.33)
Guangdong	0	4	13	13	43	27	27	26	19	17	22	28	45	39	56	39	14	432 (24.96)
Guanxi	0	0	2	2	14	9	23	12	8	8	9	13	19	6	12	16	1	154 (8.90)
Guizhou	0	0	1	0	1	5	2	8	2	9	15	6	4	6	7	10	3	79 (4.56)
Hainan	0	0	1	10	5	2	5	2	3	0	3	4	3	8	3	7		56 (3.24)
Hebei	0	0	0	5	5	3	7	1	1	3	6	4	11	2				48 (2.77)
Henan	0	0	4	5	13	5	1	0	1	2	9	7	8	4	4			63 (3.64)
Hunan	0	0	11	15	18	20	18	22	22	37	51	53	41	28	25	26	3	390 (22.53)
Jiangxi	0	0	1	12	10	4	11	5	2	5	10	2	1					63 (3.64)
Shaanxi	0	0	0	1	1	5	9	6	15	4	2							43 (2.48)
Sichuan	0	0	6	7	7	9	11	9	10	3	13	17	23	12				127 (7.34)
Yunnan	0	0	0	0	6	18	23	14	7	18	18	32	31	21	13			201 (11.61)
Total (%, n=1731)	0	4 (0.23)	40 (2.31)	79 (4.56)	127 (7.34)	125 (7.22)	144 (8.32)	112 (6.47)	92 (5.31)	108 (6.24)	160 (9.24)	173 (9.99)	188 (10.86)	132 (7.63)	123 (7.11)	103 (5.95)	21 (1.21)	1731 (100.00)

ICSRs, Individual Case Safety Reports.

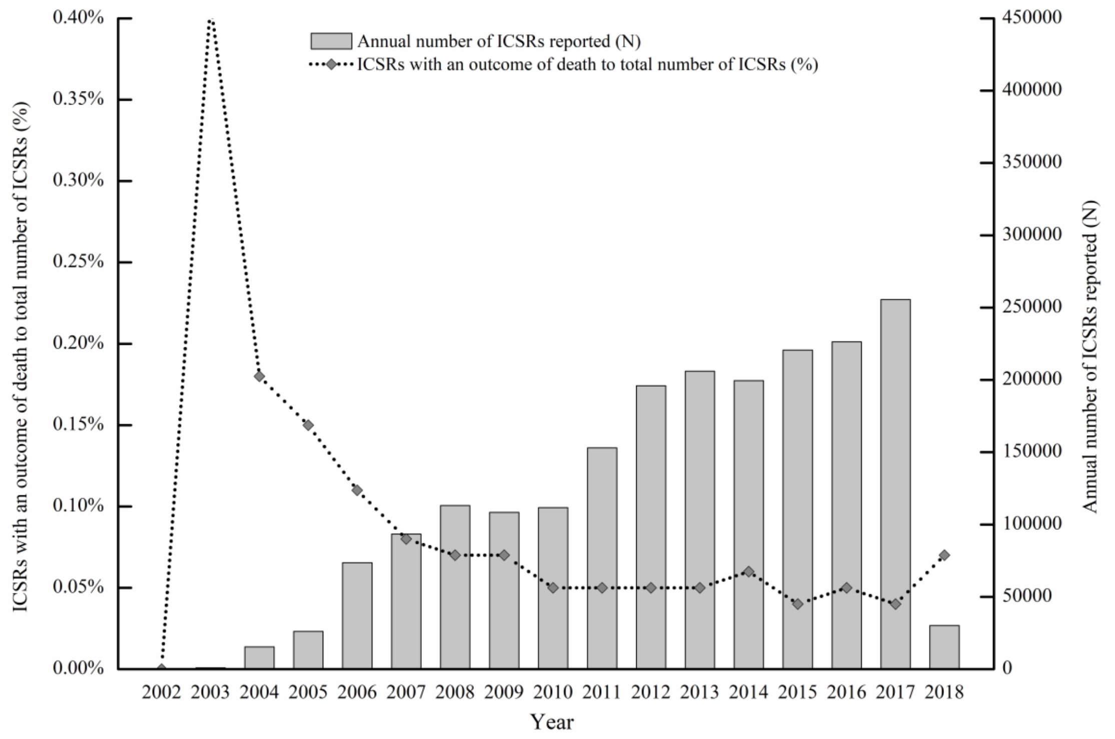

Supplementary Figure 1. Annual numbers of ICSRs reported and the percentage of death ICSRs in 5 provinces with all data available during the whole period

Supplementary Table 2. Detailed information on death ICSRs related to drug use of female reproductive disorders

Case	Age (years)	Weight (kg)	Suspected drugs	ADR(s) reported (IT)	Primary diseases (ICD-10) [a]
1	16	NA	Estradiol valerate tablet	Hepatic failure, Shock, Acidosis, Respiratory distress syndrome, Encephalopathy	Amenorrhoea, unspecified (N91.2)
2	23	55	Misoprostol tablet	Anaphylactic shock	Spontaneous abortion : complete or unspecified, without complication (O03.9)
3	25	65	Mifepristone tablet	Uterine haemorrhage	Medical abortion : complete or unspecified, without complication (O04.9)
4	28	50	Oxytocin injection+ Misoprostol tablet+ Mifepristone tablet	Palpitations, Unconsciousness	Medical abortion : incomplete, without complication (O04.4)
5	32	65	Oxytocin injection + Calcium gluconate injection	Embolism pulmonary, Disseminated intravascular coagulation	Single spontaneous delivery, unspecified (O80.9)
6	35	70	Oxytocin injection	Anaphylactic shock	Prolonged pregnancy (O48)
7	36	58	Baofukang suppository [b]	Fetal death	Infections of the genital tract in pregnancy (O23.5)
8	38	74	Oxytocin injection + Vitamin B1 injection + Calcium gluconate injection + Cefazolin sodium pentahydrate injection +Fat-suluble vitamin injection +Water-soluble vitamin injection	Anaphylactic shock	Diseases of the skin and subcutaneous tissue complicating pregnancy, childbirth and the puerperium (O99.7), Other specified noninflammatory disorders of uterus (N85.8)
9	39	48	Misoprostol tablet	Anaphylactic shock, Apnoea	Medical abortion : complete or unspecified, without complication (O04.9), Endocrine, nutritional and metabolic diseases complicating pregnancy, childbirth and the puerperium (O99.2)
10	45	48	Mifepristone tablet+ Misoprostol tablet	Anaphylactic shock	Medical abortion : complete or unspecified, without complication (O04.9)

IT, WHO-ART included terms; [a] ICD-10, International Statistical Classification of Diseases and Related Health Problems 10th Revision; NA, No information available; [b] a vaginally administered traditional Chinese medicine made from zedoary turmeric oil and Borneol, others are conventional medicines.

Supplementary Table 3. Top 10 most frequently reported ADEs and the suspected drugs

Code	System Organ Class	ADEs (PT)	No. of records	Suspected drugs (No. of ICSRs) [a]
1830	Immune disorders and infections	Anaphylactic shock	763	Cefoperazone Sodium and Sulbactam Sodium (39), Ceftriaxone Sodium (35), Iopromide (17), Benzylpenicillin (17), Benzathine Benzylpenicillin (14), Cefotaxime sodium (13), Dextran 40 (12), Qingkailing (11)[b], Lidocaine Hydrochloride (9), Cefoperazone Sodium (8), Cefuroxime Sodium (8)
1810	Body as a whole-general disorders	Death	235	Low Calcium Peritoneal Dialysis Solution (lactate) (35), Ceftriaxone Sodium (9), Iloprost Solution for Inhalation (6), Moxifloxacin Hydrochloride (4), Iopromide (4), Itraconazole (3), Xiangdan (3) [b]
1100	Respiratory disorders	Dyspnoea	111	Cefoperazone Sodium and Sulbactam sodium (5), Ceftriaxone Sodium (4), Moxifloxacin Hydrochloride (4), Qingkailing (3) [b], Ceftazidime (3)
1830	Immune disorders and infections	Anaphylactoid reaction	70	Ceftriaxone Sodium (3), Benzathine Benzylpenicillin (3), Clindamycin Phosphate (2), Benzylpencillin Sodium (2), herba houttuyniae (2)
1100	Respiratory disorders	Respiratory insufficiency	70	Itraconazole (4)
1000	Cardiovascular disorders	Cardiac arrest	64	Iopromide (8), Vinpocetine Sodium Chloride (2), Iohexol Injection(2)
1810	Body as a whole-general disorders	Sudden death	62	Diclofenac Sodium (3), Cefoperazone Sodium and Sulbactam Sodium (2), Ceftriaxone Sodium (2)
1000	Cardiovascular disorders	Cyanosis	39	Cefoperazone Sodium and Sulbactam Sodium (2), Cefathiamidine (2)
1810	Body as a whole-general disorders	Chest pain	39	Cefoperazone Sodium and Sulbactam Sodium (2), Cervus and Cucumis Polypetide (2), Moxifloxacin Hydrochloride and Sodium Chlorid (2)
0400	Neurological disorders	Muscle contractions involuntary	38	Cefoperazone Sodium and Sulbactam Sodium (2), Iopromide (2), Vinpocetine Sodium Chloride (2), Qingkailing (2)[b]

PT, WHO-ART preferred terms; [a] Dosage form is injection except Iloprost solution for inhalation; [b] TCM, others are conventional drugs.
